# Australian podiatry workforce: findings from the PAIGE cross-sectional study of Australian podiatrists

**DOI:** 10.1186/s13047-023-00646-8

**Published:** 2023-08-01

**Authors:** Anna Couch, Terry Haines, Belinda O’Sullivan, Hylton B. Menz, Cylie M. Williams

**Affiliations:** 1 Peninsula Health, Allied Health, Hastings Rd, Frankston, VIC 3199 Australia; 2 School of Primary and Allied Health Care, Monash University, 47-49 Moorooduc Hwy, Frankston, VIC 3199 Australia; 3 Frankston Integrated Health, 2 Hastings Road, Frankston, VIC 3199 Australia; 4 School of Rural Health, Monash University, Mercy St, Bendigo, VIC 3550 Australia; 5 The University of Queensland, Brisbane, QLD 4072 Australia; 6 School of Allied Health, Human Services and Sport, La Trobe University, VIC 3086 Bundoora, Australia

**Keywords:** Podiatry, Demographics, Workforce, Work patterns, Metropolitan, Rural

## Abstract

**Background:**

Understanding the dynamics of the podiatry workforce is essential for the sustainability of the profession. This study aimed to describe the podiatry workforce characteristics and identify factors associated with rural practice location.

**Methods:**

We used an exploratory descriptive design from data obtained during cross sectional study: Podiatrists in Australia: Investigating Graduate Employment through four online surveys (2017–2020). Demographic and workplace characteristics including career development were described. Univariate logistic regressions were used to determine associations with rural or metropolitan practice location.

**Results:**

Data were included from 1, 135 podiatrists (21% of *n* = 5,429). There were 716 (69% of *n* = 1,042) females, 724 (65% of *n* = 1,118) worked in the public health service and 574 (51% of 1,129) were salaried employees. There were 706 (87% of *n* = 816) podiatrists with access to paid annual leave and 592 (72% of *n* = 816) to paid sick leave. There were 87 (32% of *n* = 276) podiatrists who reported 51–75% of workload involved Medicare bulk-billed Chronic Disease Management plans, and 324 (74% of *n* = 436) not utilising telehealth. The majority of podiatrists (57% of *n* = 1,048) indicated their average consultation length was 21 -30 min, and patients typically waited < 3 days for an appointment (41% of *n* = 1,043). Univariate logistic regression identified podiatrists working in metropolitan settings have less years working in current location (OR = 0.98, 95% CI = 0.96, 0.99), less working locations (OR = 0.91, 95% CI = 0.86, 0.97), were less likely to have access to paid annual leave (OR = 0.65, 95% CI = 0.43, 0.98), and paid sick leave (OR = 0.65, 95% CI = 0.46, 0.95), shorter waiting periods for appointments (OR = 0.44, 95% CI 0.30, 0.64) and more likely to utilise telehealth within their practice (OR = 2.03, 95% CI 1.19, 3.50) than those in rural locations.

**Conclusion:**

These results provide insight into the profession uncommonly captured in workforce planning data. This included the number of working locations, billing practices and wait lists. This also highlights opportunities to promote rural training pathways, service integration to build attractive podiatry positions that are tailored to meet the needs of rural communities and solutions to make telehealth more accessible to podiatrists.

**Supplementary Information:**

The online version contains supplementary material available at 10.1186/s13047-023-00646-8.

## Background

The allied health workforce is a rapidly growing essential part of Australia’s health workforce with approximately 200,000 registered allied health professionals nationwide [1]. However, the allied health workforce remains relatively poorly described. Factors related to the geographic maldistribution and undersupply of allied health professionals in rural and remote towns is noted to need more attention [2]. Improved access to consistent and reliable workforce data has been identified as a key solution to address these shortages by informing workforce planning and policy development [2, 3].

Podiatry is a small and essential allied health profession for specialist lower limb care, with approximately 5952 registered podiatrists as of December 2022 in Australia [4]. In Australia, podiatrists work alongside medical professionals, nursing, and allied health to provide care across acute inpatient services, inpatient and outpatient rehabilitation, residential aged care, private practice and with participants of the National Disability Insurance Scheme [5]. Podiatrists’ contribution to holistic patient care, including as part of the team-based care models is increasingly being recognised within the 10 year primary care plan [6] and Strengthening Medicare Taskforce Report [7].

In Australia, approximately 65% of podiatrists work in private practice and 35% work in a public health service [8]. In the private sector, podiatry services are provided on a ‘fee for service’ model with subsidies available through Medicare, an Australian Government funded medical insurance scheme [9], or private health insurance entities (or user pay). Australians’ with a chronic health condition or complex care needs can access up to five podiatry visits through a Chronic Disease Management (CDM) plan [10]. To access this funding support, their general practitioner coordinates a team care arrangement. This funding support allows for a limited number of allied health practitioners to provide a professional service at their own fees, the Medicare Benefit Schedule can incur an out of pocket cost to the patient [10]. Some allied health professionals may bulk-bill this type of consultation, meaning the service is provided at no cost to the patient. Other funding systems that support people to access podiatry at no or low out of pocket costs include the National Disability Insurance Scheme (NDIS) [5], Home Care Packages Program [11] and state and federal government funding allocated to public podiatry health located in community health services.

The need for an increase in Australian allied health professionals has been identified by The Royal Commission into Aged Care Safety and Quality [12], NDIS National Workforce Plan 2021–2025 [13], The Rural Health Commissioner’s Report [2], the Productivity Commission in its inquiry into Mental Health [14], and more recently Australia’s Primary Health Care 10 year plan [6] and Strengthening Medicare Taskforce Report [7]. Multiple labour force surveys have been undertaken worldwide to inform future policy directions and allied health workforce strategies [15, 16] but there is little consistency in how the data has been collected and integrated [17]. The National Rural Health Commissioner provided detailed recommendations regarding focused investment in allied health data, specifically highlighting the need for the development of a National Allied Health Data Strategy and a National Allied Health Workforce Minimum Dataset [2, 17]. Within these recommendations there was an identified need for data to support workforce planners to see more of a complete picture of the allied workforce including student placements, understanding how clinicians work (in dual roles/or across different sectors) and models of care delivery (e.g. telehealth, face-to-face or outreach). The recommendation provided by the National Rural Health Commissioner also outlined the importance of reviewing workforce dynamics based on rurality [18].

The Podiatry Board of Australia publishes registrant data quarterly [4] and the Australian Institute of Health and Welfare [19] collects some workforce data but it is limited to basic demographics and distribution by state and territory. There are no national studies of the Australian podiatry workforce. The Podiatrists in Australia: Investigating Graduate Employment (PAIGE) study was designed to create a unique national podiatry workforce dataset, and more specifically to understand why Australian podiatrists choose to work where they do. The primary aim of this study was to describe podiatry workforce features with secondary aims to identify any differences that may be related to rurality.

## Methods

### Design

This study was an exploratory descriptive study using data from the Podiatrists in Australia: Investigating Graduate Employment (PAIGE) cross sectional study. The data were collected through four online surveys between 2017 and 2020. Approval was given by the Monash University Human Research Ethics Committee (approval number 19959). The CHERRIES (Checklist for Reporting Results of Internet E-Surveys) guided the reporting of collected data [20].

### Participants and setting

Waves 1 and 2 of the survey (2017–2018) was open to podiatrists and podiatric surgeons living in Victoria and waves 3 and 4 (2019–2020) was open to podiatrists and podiatric surgeons Australia wide. When the fourth wave of the survey closed, there were an estimated 5,429 podiatrists and 36 podiatric surgeons registered in Australia [21]. Participants were recruited each year through directed emails to members of peak bodies and special interest groups such as the Australasian College of Podiatric Surgeons, Advanced Practicing Podiatrist- High Risk Foot Group, Podiatry Western Australia, and the Australian Podiatry Association. The survey was also promoted on social media (Facebook, Twitter, LinkedIn, and Instagram), podiatry conferences across Australia and public health networks. If a participant had completed a previous survey wave and left their contact details, they were directly invited to complete the next wave via email. Competition based incentives were used in the recruitment process, with participants having the option to enter a draw at the completion of the survey to win a $100 (AUD) voucher to be used for professional development through the Australian Podiatry Association.

### Data collection

Data were collected as per the published methodology [22] as part of the PAIGE study. The PAIGE study survey tools were based on the Medicine in Australia: Balancing Employment and Life (MABEL) study [23] with the primary aim of understanding intrinsic and extrinsic factors that impact the podiatry workforces labour decisions [24]. Questions were modelled on the MABEL study using similar wording for a core set of demographic data domains, in addition to different question bank elements added into subsequent waves. Details of the PAIGE study, data analysis methods and data domains collected are published in detail elsewhere [8]. Each four waves of the survey are provided as Supplementary Files 1, 2, 3 and 4.

Demographic data collected from podiatrists included information about their age, gender, recency of practice, primary work setting, business relationship, working locations, time spent working at a location, if they undertook a regional/rural placement during their training, if they grew up in a rural area and their overall health rating.

Workplace characteristic data collected from podiatrists included information about the hours worked per week, number of other podiatrists and health professionals in their primary workplace, leave entitlements, waiting periods for patients, average consultation time, billing structures and workload percentages relating to Medicare Chronic Disease Management plan patients, telehealth consultations, National Disability Insurance Scheme participants and aged care work.

Industry led career education data collected from podiatrists included information about their plans to apply for Australian Podiatry Association Career Framework in Paediatric, Sports, High Risk Foot or to undertake surgical training within the Australian College of Podiatric Surgeons, and intentions to seek endorsement on their registration for use of scheduled medicine based on the requirements set by the Podiatry Board of Australia.

### Procedure

Survey data of each wave were collected through Qualtrics® software (Qualtrics, Provo, UT, USA) [25]. Participants created their own consistent and unique identifier code so that responses could be linked in subsequent waves and unknown to the research team. If it was the participants first time completing the survey (in any year), all questions were asked. If a podiatrist had participated in a previous wave and had not changed job or living location, the survey software used question logic and demographics such as gender, age and year of graduation were shown to ensure accurate linkage in addition to new questions added into the new wave of questions. To minimise missing data, forced or requested response prompts were used, but podiatrists could withdraw at any time by closing their internet browser. For partial completion, cookies were used to allow responses to be saved up to 4 h. Internet Protocol (IP) addresses are routinely collected by Qualtrics® as the de-identified metadata in the survey responses. Where linking variables were incomplete, IPs were viewed and used as a last resort to match data.

### Analysis

Data were initially cleaned to remove any responses that did not include core demographics (age, gender, postcode, recency of practice). A final per podiatrist response was created with the most recent response, inputting data from previous waves where required for podiatrists who had completed more than one of the survey waves.

Stata 15 software (StataCorp, College Station, TX, USA) was used to analyse data. Descriptive statistics of all variables were used to report on each variable of interest for the entire cohort and then grouped based on postcode data into metropolitan responses (MMM 1) or rural responses (MMM 2, 3, 4, 5, 6, and 7) using the Modified Monash Model (MMM) [26].

Univariate logistic regression was used to determine any characteristics that were independently associated with rural or metropolitan work location. Work location was used as a binary dependent variable in logistic regression analysis where the resultant odds ratio results were presented and can be interpreted as the change in odds in a 1 unit change in the predictor variables.

## Results

### Participant characteristics

There were 1,135 podiatrists who completed the majority of the demographic questions in at least one of the survey waves. At the closure of wave 4 (2020) this response rate was estimated to be 21% (1,135 of 5,429) of the Australian podiatry workforce [21]. Participant demographics were similar to those reported in Australian podiatry registrant data [21]. Table 1 displays a breakdown of podiatrists’ demographics, work setting, employment profile, regional/rural exposure, and overall health rating. Mean (SD) age of podiatrists were 39 (11) and 69% identified as female. Of the total number (1,118) of podiatrists, 724 (65%) primary work setting was within a private practice. Over half (51% of 1,118) were salaried employees and the mean (SD) working locations was 2.3 (2). Of the 1,048 podiatrists who responded, 389 (37%) reported their health as ‘excellent’, 411 (39%) as ‘very good’ and 198 (19%) as ‘good’.

**Table 1:** Demographics of participants

	**Total responses *n* = 1,135(100%) Mean (SD**^**a**^**) or *Median (IQR***^**b**^***)***	**Metro responses *n* = 796 (70%) Mean (SD) or *Median (IQR)***	**Rural responses *n* = 339 (30%) Mean (SD) or *Median (IQR)***	**Odds Ratio (OR), 95% Confidence Interval (CI)**
**Age**	***n* = 1,045**	***n* = 726**	***n* = 319**	
(years)	39(11)	39(11)	39(11)	1.0(0.99–1.01)
**Gender**	***n* = 1,042**	***n* = 725**	***n* = 317**	
Did not identify as female	326 (31%)	242 (33%)	84 (26%)	Reference group
Female	716 (69%)	483 (67%)	233 (74%)	0.82(0.62–1.10)
**Recency of practice**	***n* = 977**	***n* = 683**	***n *= 294**	
(years)	14(11)	14(11)	14(12)	0.99(0.99–1.01)
**Primary work setting**	***n* = 1,118**	***n* = 784**	***n* = 334**	
Private practice	724 (65%)	516(66%)	208(62%)	Reference group 1.19(0.91–1.54)
Public health service	394 (35%)	268(34%)	126(38%)	
**Business relationship**	***n* = 1,129**	***n* = 791**	***n* = 338**	
Owner or partner	330 (29%)	228 (29%)	102(30%)	Reference Group
Salaried employee	573 (51%)	394 (50%)	179(53%)	0.98(0.73–1.32)
Contracted employee	180 (16%)	137 (17%)	43(13%)	1.42(0.94–2.15)
Locum/Not working	14 (1%)	10 (1%)	4(1%)	1.11(0.34–3.63)
Other	32 (3%)	22 (3%)	10(3%)	0.93(0.44–1.98)
**Working locations**	***n* = 1,135**	***n* = 796**	***n* = 339**	
(number)	2.3(2)	2.2(2)	2.6(2)	0.91(0.86–0.97)
**Time working in this location**	***n* = 1,057**	***n* = 732**	***n* = 327**	
(years)	*4(1,11)*	*4(1,14)*	*5 (1,14)*	0.98(0.96–0.99)
**Regional/rural placements**	***n* = 693**	***n* = 435**	***n* = 196**	
(yes)	231 (33%)	160 (37%)	71 (36%)	1.72(1.28–2.32)
**Grew up in a rural area**	***n* = 236**	***n* = 163**	***n* = 73**	
(yes)	102 (43%)	54 (34%)	48 (66%)	0.26(0.14–0.46)
**Overall health rating**	***n* = 1,048**	***n* = 728**	***n* = 320**	
Excellent	389 (37%)	285 (39%)	104 (32%)	Reference group
Very good	411(39%)	276 (38%)	135 (42%)	0.75(0.55–1.01)
Good	198 (19%)	133 (18%)	65 (20%)	0.75(0.51–1.08)
Fair	37 (4%)	25 (3%)	12 (4%)	0.76(0.37–1.57)
Poor	13 (1%)	9 (1%)	4 (1%)	0.82(0.25–2.72)

^a^Standard deviation

^b^Interquartile range

### Workplace characteristics

The median (IQR) of hours worked per week by podiatrists was 26 (4, 38) hours and the median (IQR) number that each participant worked with in their main workplace was 2 (1,4) female podiatrists and 2 (1,3) male podiatrists (Table 2). Of the 816 podiatrists who responded to this question, 706 (87%) had access to paid annual leave, 521 (80%) had access to unpaid annual leave, 592 (72%) had access to paid sick leave and 177 (32%) had no leave available. There were 423 (41% *n* = 1043) report their patients typically wait < 3 days for an appointment.

**Table 2:** Workplace characteristics

Workplace characteristics		Total responses *n* = 1,135 (100%) Mean (SD) or *Median (IQR)*	Metro responses *n *= 796 (70%) Mean (SD) or *Median (IQR)*	Rural responses *n* = 339 (30%) Mean (SD) or *Median (IQR)*	Odds Ratio (OR), 95% Confidence Interval (CI)
**Hours worked per week**		***n* = 1,072** *26(4, 38)*	***n* = 745** *25(4, 38)*	***n* = 327** *24(4,38)*	1.00(0.99–1.00)
**Female podiatrists in** **main workplace**	(number)	2(2**( *n* = 1,035** *2(1,4)*	***n* = 721** *3(1,4)*	***n *= 314** *2(1, 4)*	1.01(0.95–1.08)
**Male podiatrists in main workplace**	(number)	***n* = 903** *2(1,3)*	***n* = 636** *2(1, 3)*	***n* = 267** *2(1,3)*	1.05(0.94–1.18)
**Allied health professionals in main workplace**	(number)	***n* = 895** *3(2,6)*	***n* = 626** *4(2,6)*	***n* = 269** *3(2,6)*	1.00(0.99–1.02)
**Other health professionals in workplace**	(number)	***n* = 982** *3(0,15)*	***n* = 685** 5(1,20)	***n* = 297** *2(0,15)*	0.99(0.99–1.00)
**Paid annual leave**	(yes)	***n* = 816** 706 (87%)	***n* = 577** 471 (81%)	***n* = 239** 235 (98%)	0.65(0.43–0.98)
**Unpaid annual leave**	(yes)	***n* = 816** 521(80%)	***n* = 577** 376 (73%)	***n* = 239** 145(61%)	1.28(0.91–1.82)
**Paid sick Leave**	(yes)	***n* = 816** 592 (72%)	***n* = 577** 380 (72%)	***n* = 239** 190 (79%)	0.65(0.46–0.95)
**No Leave available**	(yes)	***n* = 816** 177 (22%)	***n* = 577** 127 (22%)	***n* = 239** 41 (17%)	1.5(0.99–2.30)
**How many days does a patient typically wait for an appointment?**		***n* = 1043**	***n* = 726**	***n* = 317**	
	< 3 days	423 (41%)	322(45%)	101(32%)	Reference value
	4–7 days	279 (27%)	186(25%)	93(29%)	0.63(0.45–0.88)
	7–14 days	178 (17%)	123(17%)	55(17%)	0.70(0.48–1.03)
	> 15 days	163 (16%)	95(13%)	68(21%)	0.44(0.30–0.64)
**Bulkbill Medicare CDM plan**		***n* = 1,020**	***n* = 709**	***n* = 311**	
	No/don’t accept	635 (62%)	439(62%)	196(63%)	Reference value
	yes	385 (38%)	270 (38%)	115(37%)	1.05(0.79–1.38)
**% of workload involves Medicare CDM plans that are bulk billed**		***n* = 276**	***n* = 197**	***n* = 79**	
	< 25%	51 (18%)	38(19%)	13 (16%)	*Reference value*
	25–50%	65 (24%)	47(24%)	18 (23%)	0.91(0.40–2.09)
	51–75%	87 (32%)	56(29%)	31 (39%)	0.62(0.29–1.33)
	> 75%	72 (26%)	55(28%)	17 (22%)	1.11(0.48–2.54)
**% of your clinical load involves telehealth consultations?**		***n* = 436**	***n* = 310**	***n* = 126**	
	0%	324 (74%)	219(71%)	105 (83%)	*Reference value*
	1–24%	105 (24%)	85 (28%)	20 (16%)	2.03(1.19–3.50)
	> 25%	7 (2%)	6 (1%)	1 (1%)	2.88(0.34–24.20)
**Registered or accept patients who have NDIS funding**		***n* = 811**	***n* = 567**	***n* = 244**	
	(yes)	374 (46%)	253 (45%)	121 (50%)	0.82(0.61–1.11)
**% of clinical load involves assessing/treating patients with NDIS funding**		***n* = 381**	***n* = 249**	***n* = 119**	
	< 25%	365 (96%)	235 (94%)	117 (98%)	Reference value
	> 25%	16 (3%)	14 (6%)	2 (2%)	3.48(0.78–15.59)
**% of clinical load involves care provided in patients home/residential aged care facility?**		***n* = 899**	***n* = 610**	***n *= 289**	
	< 50%	842 (94%)	565 (93%)	277 (96%)	Reference value
	50–99%	35 (4%)	30 (5%)	5 (2%)	2.94(1.13–7.66)
	100%	22 (2%)	15 (2%)	7 (2%)	1.42(0.42–2.61)
**Average consultation length**		***n* = 1,048**	***n* = 730**	***n* = 318**	
	< 10 min	9 (1%)	8 (1%)	2 (1%)	Reference value
	11–15 min	25 (2%)	15 (2%)	10 (3%)	0.37(0.07–2.14)
	12–60 min	187 (18%)	127 18%)	60 (19%)	0.53(0.11–2.57)
	21–30 min	604 (57%)	410 (56%)	194 (60%)	0.53(0.11–2.51)
	> 31 min	222 (22%)	170 (23%)	52 (16%)	0.82(0.17–9.97)

^*^Standard deviation

^**^Interquartile range

### How podiatrists work

Podiatrists were asked questions related to their workload, a total of 635 (62% of 1,020) podiatrists did not accept or did not bulkbill a Medicare CDM plan and 324 (74% of 436) did not utilise telehealth consultations (Table 2). Whilst more than half of participants did not accept of bulkbill a Medicare CDM plan, 72 (26% of 276) indicated that bulkbilled Medicare CDM plans made up more than 75% of their workload. A total of 374 (46% of 811) podiatrists identified that they were registered (with the National Disability Insurance Scheme) or accepted patients who were funded through this scheme, however, 36a5 (96% of 381) podiatrists reported that assessing/treating patients with National Disability Insurance Scheme funding made up < 25% of their workload. Podiatry care delivered to patients at home, or in residential aged care facilities made up less than 50% of workload for the majority of podiatrists (94% of 899). The average consultation length was 21 to 30 min for 604 (57% of *n* = 1,048) of podiatrists who completed the survey.

### Industry led career education and progression

Podiatrists were asked to identify their intentions to pursue industry-led career education (Table 3). Of the total number of respondents (*n* = 1,135), 175 (15%) indicated that they were planning to apply to progress through the Australian Podiatry Association Career Framework, with a further 398 (35%) being unsure. These podiatrists indicated interest in a paediatric credential (*n* = 163, 32% of *n* = 504), sports podiatry (*n* = 278, 48% of *n* = 580) or high risk foot (*n* = 270, 47% of *n* = 580).

**Table 3:** Career development

		**Total responses *n* = 1,135 (100%)**	**Metro responses *n* = 796 (70%)**	**Rural responses *n* = 339 (30%)**	**Odds Ratio (OR), 95% Confidence Interval (CI)**
**Intention to apply for Australian Podiatry Association Career Framework**		***n *= 1,135**	***n* = 796**	***n *= 339**	
	Yes	175 (15%)	120 (15%)	55 (16%)	Reference value
	Unsure	398 (35%)	277 (35%)	121 (36%)	1.05(0.71–1.54)
	No	562 (50%)	399 (50%)	163 (48%)	1.12(0.78–1.62)
**Which of the following have you considered, applied for, enrolled in or waiting to commence?**		***n* = 504**	***n* = 343**	***n* = 161**	
	Paediatric	163 (32%)	104 (30%)	59 (37%)	0.75(0.51–1.11)
		***n* = 580**	***n* = 399**	***n* = 181**	
	Sports	278 (48%)	189 (47%)	89 (49%)	0.93(0.65–1.32)
		***n* = 580**	***n* = 580**	***n* = 580**	
	High Risk Foot	270 (47%)	180 (31%)	90 (16%)	0.79(0.56–1.13)
		***n* = 458**	***n* = 312**	***n* = 146**	
	Podiatric Surgery	82 (18%)	57 (18%)	25 (17%)	1.08(0.64–1.82)
**Intention on undertaking the requirements to become an endorsed prescriber**		***n* = 467**	***n* = 328**	***n* = 139**	
	Yes	58 (12%)	44 (13%)	14 (10%)	Reference value
	Unsure	103 (22%)	71 (22%)	32 (23%)	0.71(0.34–1.47)
	No	257 (55%)	175 (53%)	82 (59%)	0.68(0.35–1.31)
	In the process	27 (6%)	20 (6%)	7 (5%)	0.91(0.32–2.60)
	Already endorsed	22 (5%)	19 (6%)	4 (3%)	1.43(0.41–4.94)

There were 82 (18% of 458) podiatrists indicate they were considering further training through the Australian College of Podiatric Surgeons. There were 58 (12% of 467) indicating willingness to start the process of endorsement for schedule medicines as set out by the Podiatry Board of Australia, 103 (22% of 467) were unsure and 257 (55% of 467) did not plan to undertake the requirements. There were 27 (6% of 467) already undertaking the pathway for endorsement and 22 (5% of 467) already endorsed to prescribe at the time they completed the survey.

### Job characteristics related to rurality

Univariate logistic regression identified several significant factors related to rural compared with metropolitan work including number of working locations, time spent working in current location, access to leave (paid and sick), waiting time for appointments and telehealth utilised in clinical load (Tables 1 and 2). Podiatrists who worked in metropolitan locations had less working locations compared to podiatrists working in rural locations (OR = 0.91, 95% CI = 0.86, 0.97). Metropolitan practicing podiatrists had less years working in current location compared to those working in rural settings (OR = 0.98, 95% CI = 0.96, 0.99). Podiatrists who worked in metropolitan locations are less likely to have access to paid annual leave (OR = 0.65, 95% CI = 0.43, 0.98) and paid sick leave (OR = 0.65, 95% CI = 0.46, 0.95) (Table 2). Podiatrists indicated that patients were more likely to wait longer for an appointment (> 15 days) in rural locations (OR = 0.44, 95% CI 0.30, 0.64). Podiatrists working in metropolitan locations were more likely to utilise telehealth (1–24% of workload) compared to podiatrists working in rural locations (OR = 2.03, 95% CI 1.19, 3.50).

## Discussion

This data provides new insights into the Australian podiatry workforce, characteristics of where they work and who they work with, preferred funding models and individual career aspirations. The PAIGE study is the largest series of podiatry workforce surveys ever undertaken and provides a systematic and rigorous evidence base for podiatric workforce policy development. This data complements publicly accessible workforce data through its capacity to investigate associations and dynamics related to current practice context and rurality. This study also identified key characteristics future workforce planners should utilise to understand the profession, such as how podiatrists work (e.g. work locations, characteristics of job), how patients utilise alternative funding sources and if podiatrists want to extend their scopes of practice through the Australian Podiatry Association Career Framework and endorsement for scheduled medicines. Differences between podiatrists working locations could be used to promote rural practice to podiatrists.

Podiatrists working in rural areas had more working locations compared to podiatrists in metropolitan settings. This finding could indicate the demand on rural practicing podiatrists to split their workload across multiple settings (public, private and outreach) to meet the needs of the population groups living in these areas. Finding podiatrists who worked rurally were more likely to have worked in the same location for a greater number of years compared to those in metropolitan locations is at odds with previous research of high rural turnover [27]. Our results may reflect the older or settled demographic as the average age of podiatrists completing the survey was 39 years, with average of 14 years working years. Connection to a local and professional community, including the ability of the location to meet their (and any family members) future needs, has been linked to intention to stay working within that location long term [28]. This highlights opportunities for rural training pathways to increase exposure to rural practice and should continue to be a key workforce strategy. Especially as this exposure has been linked to an increased likeliness of working rurally in the first year of practice [2] and thereby reducing any health disparities seen between rural and metropolitan locations [29].

The podiatry workforce plays a large role in preventative healthcare, supporting general practitioners to manage foot disorders and mitigating escalation to high-risk foot clinics, reducing emergency department presentations and hospital admissions [30]. Understanding how patients fund podiatry services within private practice is fundamental to ensuring sustainability of a profession that is primarily privately funded. Changes in Medicare, disability and aged care funding models will continue to require podiatrists to understand how to support patients to access this funding. Podiatry is the most utilised Medicare-subsidised individual allied health service and patients with diabetes are the most likely to utilise an allied health service under a CDM plan [31]. This was reflected by over a quarter of podiatrists reporting that Medicare bulk-billed Chronic Disease Management plans made up > 75% of their workload. There is ongoing reform to the Medicare system in Australia and whilst its introduction removed one of the main barriers to accessing podiatry services [32], podiatrists who only bulkbill are at the behest of associate political drivers and funding reforms. This may impact business sustainability.

This is the first known study to explore podiatry utilisation of National Disability Insurance Scheme funding. Since the introduction of NDIS by the Australian Government in 2013, the allied health workforce has had to adapt to the changing landscape of consumer-controlled funding to provide support for people with disability [33]. There were limited differences between participants from metropolitan and rural locations regarding NDIS, this is despite concerns around the rural workforce’s ability to adopt the NDIS due to capacity to meet the increase in demand [34, 35]. The administrative burden, ongoing funding reforms and challenges of accessing funding is consistent across locations, meaning these findings provide a baseline for future utilisation. Ongoing allied health workforce shortages places National Disability Insurance Scheme participants at risk of having inadequate access to appropriate health service providers, more so in rural settings [35]. There may be opportunities such as those identified by the Rural Health Commissioner [2] to improve services through structured inter-professional collaboration appropriate for participants with this funding [2]. This may be an opportunity for the podiatry workforce to increase services within rural locations.

There are several limitations that should be considered when interpreting the findings of this study. Whilst the participant demographics are similar to those reported in registration data [21] and the data provides an accurate representation of the survey participants (21% of the Australian podiatry workforce), results may not be generalisable to the entire Australian podiatry workforce. Whilst this data is retrospective in nature, our results are one of the largest collected from Australian podiatrists and workforce data is essential for government entities to make funding decisions and workforce recommendations [24]. It is unlikely that any one domain was subject to self-selection bias due to the generalised nature of the wording of the questions. The timeframe of data collection was also pre-COVID-19 pandemic. Since this time there is significant model of care and funding reforms within Australia that impacts the data, in particular the use of telehealth.

Whilst this research provides detailed data on the podiatry workforce, we recognise that there are future research opportunities. Whilst the by-line “foot experts” is commonly used to promote the podiatry profession, we are still unclear on what podiatrists do on a daily basis and how this varies between settings. This challenge extends beyond podiatry, and the development of a dataset of elements to collect data on what different allied health professionals do from day to day would be extremely beneficial to address gaps in knowledge for future workforce planning. Lastly, researchers should consider collecting more detailed data on the service provision of telehealth, National Disability Insurance Scheme and patients who have a Medicare Chronic Disease Management plan including for which health conditions, pathology and what treatment modalities are used and their effectiveness.

## Conclusion

Podiatry is a small but essential allied health profession and understanding how and where podiatrists work, who they work with, their utilisation of different funding models and opportunities for ongoing education is fundamental for future workforce planning. Podiatrists who participated in the survey were more likely to be female, work in private practice and be a salaried employee. On average, podiatrists worked at more than 1 location with at least 2 other colleagues (other podiatrist or health professional). Most podiatrists had access to annual leave, did not accept or bulkbill a (CDM) plan and did not utilise telehealth within their workload. Rural podiatrists indicated that they had worked in that location for longer than metropolitan podiatrists but had more working locations. Wait times in rural locations were perceived to be higher, highlighting concerns of unmet needs of rural populations. Workforce planners should continue to promote rural training pathways to expose podiatry students to rural practice, service integration to build attractive podiatry positions that are tailored to meet the needs of rural communities and solutions to make telehealth more accessible to podiatrists. Whilst this study was the first to explore the workload of podiatrists, further research is required to understand what podiatrists see and treat on a day-to-day basis (health conditions, treatment modalities and funding models).

## Supplementary Information

**Additional file 1. **

**Additional file 2. **

**Additional file 3. **

**Additional file 4. **

## Data Availability

Request for further details of the data set and queries relating to data sharing arrangements may be submitted to Cylie Williams (cylie.williams@monash.edu). Aggregate or summarised data may be shared based on reasonable request.

## Abbreviations

PAIGE: Podiatrists in Australia: Investigating Graduate Employment
IP: Internet Protocol
CDM: Chronic Disease Management Plan
NDIS: National Disability Insurance Scheme
CHERRIES: Checklist for Reporting Results of Internet E-Surveys
AUD: Australian Dollars
MABEL: Medicine in Australia: Balancing Employment and Life
MMM: Modified Monash Model
IQR: Interquartile range
SD: Standard Deviation
OR: Odds Ratio
CI: Confidence Interval
